# Modulating Multiarticular Energy during Human Walking and Running with an Unpowered Exoskeleton

**DOI:** 10.3390/s22218539

**Published:** 2022-11-06

**Authors:** Tiancheng Zhou, Zhijie Zhou, Hanwen Zhang, Wenbin Chen

**Affiliations:** State Key Laboratory of Digital Manufacturing Equipment and Technology, School of Mechanical Science and Engineering, Institute of Medical Equipment Science and Engineering, Huazhong University of Science and Technology, Wuhan 430074, China

**Keywords:** energy modulation, metabolic energy, unpowered exoskeleton, walking, running

## Abstract

Researchers have made advances in reducing the metabolic rate of both walking and running by modulating mono-articular energy with exoskeletons. However, how to modulate multiarticular energy with exoskeletons to improve the energy economy of both walking and running is still a challenging problem, due to the lack of understanding of energy transfer among human lower-limb joints. Based on the study of the energy recycling and energy transfer function of biarticular muscles, we proposed a hip–knee unpowered exoskeleton that emulates and reinforces the function of the hamstrings and rectus femoris in different gait phases. The biarticular exo-tendon of the exoskeleton assists hamstrings to recycle the kinetic energy of the leg swing while providing hip extension torque in the swing phase. In the following stance phase, the exo-tendon releases the stored energy to assist the co-contraction of gluteus maximus and rectus femoris for both hip extension and knee extension, thus realizing the phased modulation of hip and knee joint energy. The metabolic rate of both walking (1.5 m/s) and running (2.5 m/s) can be reduced by 6.2% and 4.0% with the multiarticular energy modulation of a hip–knee unpowered exoskeleton, compared to that of walking and running without an exoskeleton. The bio-inspired design method of this study may inspire people to develop devices that assist multiple gaits in the future.

## 1. Introduction

Walking and running are the most common gaits in human daily life. In order to enhance human mobility and augment human performance during walking and running, lots of attempts have been made to develop exoskeletons to reduce the metabolic rate of walking and running. In the past few years, significant advances have been made in developing separate exoskeletons to enhance human mobility by reducing the metabolic rate of human walking or running [1]. Autonomous powered exoskeletons reduce the metabolic rate of walking [2,3,4,5] or running [6] by partly replacing the positive joint power with the net mechanical power input in specific gait phases. Although the biological joint power can be reduced by the substantial mechanical power of the powered exoskeleton, the exoskeleton mass will also cause metabolic penalty [7], which will partly offset the effect of the assistance [8]. Researchers have to make a trade-off between the actuation duration and metabolic penalty of the exoskeleton mass [9]. Most powered exoskeletons that can reduce the metabolic rate of running are tethered powered exoskeletons [10]. As alternatives, unpowered exoskeletons only exploit lightweight passive elements to assist humans in recycling energy efficiently and thus reduce the metabolic rate of walking [11,12,13,14,15] or running [16,17]. However, most exoskeletons are designed to modulate the energy of lower limbs based on the biomechanics of a certain gait (walking or running).

After breakthroughs in the studies of exoskeletons for walking or running assistance, researchers have attached more significance to the versatility of exoskeletons and have tried to develop exoskeletons to simultaneously reduce the metabolic rate in multiple gaits. The powered autonomous hip exosuit [6], which provides humans with customized, biologically scaled [18] and simulation-optimized [10] hip extension torque profiles during walking and running based on the online detection of human COM (center of mass) fluctuations [19], can reduce the metabolic rate by 9.3% and 4.0%, respectively. In our previous work, the hip unpowered exoskeleton [20], which assists hip flexors to recycle energy in the common energy consumption period of both gaits, was demonstrated to reduce the metabolic rate by 7.2% and 6.8% during walking (1.5 m/s) and running (2.5 m/s), respectively. Although these two exoskeletons were demonstrated to reduce the metabolic rate by modulating single-joint power, the modulation of multiarticular energy to improve the energy economy during both gaits is still a challenging problem.

Several crucial factors are hindering the development of a multiarticular exoskeleton for both walking and running assistance. One of the crucial factors is the fundamental differences in biomechanics between walking and running [6,21]. Although humans can easily switch from walking like an inverted pendulum [22] to a more bouncing gait of running [23] with increasing locomotion speed, the biomechanics of lower limbs show significant differences between the two gaits in various aspects, such as muscle behaviors [24,25], the ratio of lower-limb joint powers [26] and center of mass (CoM) fluctuation characteristics [21]. The differences in biomechanics between walking and running may result in the failure to reduce the metabolic rate when applying effective assistive principles for walking (running) to running (walking). The ankle unpowered exoskeleton [11], which exploits a clutch-spring mechanism to assist ankle muscles–tendons in recycling energy efficiently, was demonstrated to reduce the metabolic rate by 7.2% during walking. On the contrary, the authors found that the metabolic rate of running increased by 11.1% with the same passive assistance provided by an ankle exoskeleton emulator [27]. Similarly, the unpowered hip exoskeleton [16], which exploits a torsional spring to recycle mechanical energy during hip flexion to assist the hip extension of the contralateral hip joint during the swing phase, was demonstrated to reduce the metabolic rate of running by 8%. When this coupled assistance method was applied to walking, the authors found the metabolic rate could not be reduced, possibly due to the fact that hip extension assistance has a negative effect on the biomechanics of hip joints during leg swing [16]. Moreover, multi-articular assistance is more likely to interfere with the natural biomechanics during both walking and running. A multiarticular unpowered exoskeleton [9] was designed to provide ankle plantarflexion and hip flexion assistance in a coupled manner using a multiarticular exo-tendon. Although the biological joint torque of participants could be reduced with assistance, the authors found that the ankle joint moment was also significantly altered, which was the main reason why the metabolic rate could not be reduced with assistance [9]. More recently, studies regarding multiarticular powered [28] and (quasi-)unpowered exoskeletons [15,29] showed that the biological effort and metabolic rate of walking can be reduced by assisting energy transfer among joints. However, these multiarticular exoskeletons have not been applied to running and have not been proved to reduce the metabolic rate of running. A more complex structure of an multiarticular exoskeleton will cause more metabolic penalties than that of a monoarticular exoskeleton, especially during running. The metabolic penalty coefficients of added mass during running are 1.4–4.4 times higher during walking (summarized in supplementary materials of [6]). So, the metabolic penalty is also a key factor that should be considered when designing a multiarticular exoskeleton.

As mentioned above, the development of an exoskeleton to modulate multiarticular energy during both walking and running is still a challenging problem. In this paper, the energy-saving properties of biarticular muscles were analyzed first. Inspired by the energy recycling and energy transfer function of biarticular muscles, we proposed a hip–knee unpowered exoskeleton that exploits exo-tendons to realize the phased modulation of multiarticular energy during both walking and running. To evaluate the assistance effect and human responses, we compared and statistically analyzed the metabolic rate, muscle activities, kinetics and kinematics of walking/running with assistance to that of walking/running without an exoskeleton.

## 2. Design and Implementation of an Unpowered Exoskeleton for Multiarticular Biomechanical Energy Modulation

### 2.1. Energy Transfer Function of Lower-Limb Biarticular Muscles

**Figure 1:**
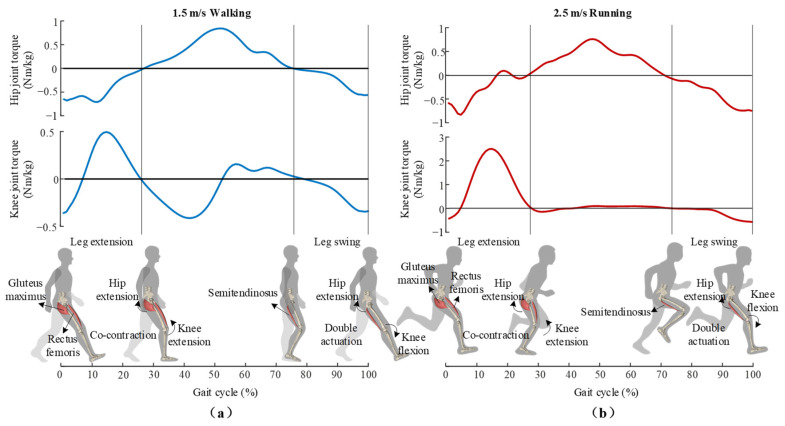
Joint torque and actions of biarticular muscles during (**a**) walking and (**b**) running. The semitendinosus (biarticular muscle) store part of kinetic energy during leg swing to provide hip with extension torque. The gluteus maximus (biarticular muscle) and rectus femoris (biarticular muscle) co-contract during leg extension.

During human locomotion, the biarticular muscles play an important role in enhancing energy economy [30,31]. As shown in Figure 1, in the late swing phase, the hamstring muscles perform negative mechanical work on the knee joint to recycle the kinetic energy of the leg swing, while providing hip joint with extension torque. The double actuation of hamstrings enables hip joint to utilize the recycled energy simultaneously [32,33]. In the following stance phase, the active contraction of the gluteus maximus actuates hip extension, thus pulling the human body forward. During this phase, the rectus femoris co-contracts with the gluteus maximus. As the rectus femoris is a biarticular muscle that actuates both hip flexion and knee extension, the power produced by the gluteus maximus (mono-articular hip extensor) can be transferred via the rectus femoris to the knee joint, and thus provide net torque on the knee joint [34,35,36]. The powerful muscle group (the gluteus maximus) can contribute to the net extension torque of the knee joint by such mono-articular and bi-articular muscle coupling, which is also considered an effective way to enhance the energy economy of human locomotion [36]. Moreover, in the process of movement, biarticular muscles can also transfer energy between different gait phases. Rectus femoris and hamstring muscles work like biological tendons undergoing stretching–shortening cycles and promoting energy recycling–releasing mechanisms during walking and running [37,38]. The previous model analysis showed that the mechanical work cost of walking and running can be reduced by 11.6% [39] and 34% [33] with the activation of both mono-articular and biarticular muscles compared to the activation of monoarticular muscles alone. On the one hand, the energy transfer among lower-limb joints during different gait phases of bi-articular muscles, as well as mono-articular and bi-articular muscle coupling, is considered an energy-saving mechanism. On the other hand, it also can be an inspiration for unpowered exoskeletons which emulate and reinforce the energy transfer function to further improve the energy economy of human walking and running.

### 2.2. Phased Multiarticular Energy Modulation of Hip–Knee Unpowered Exoskeleton

**Figure 2:**
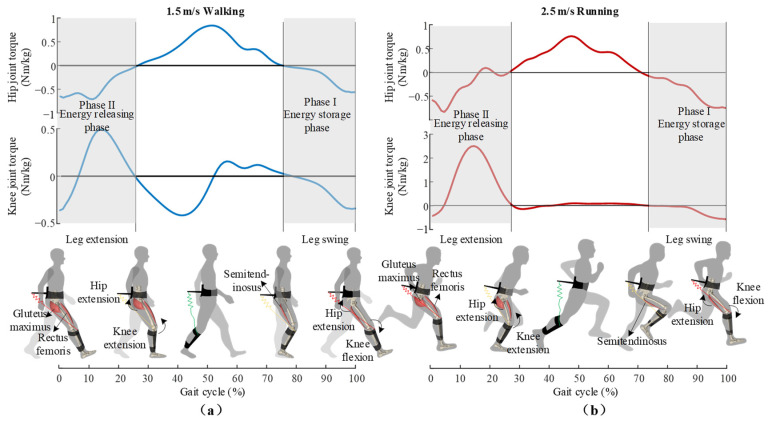
Working process of the exoskeleton during (**a**) walking and (**b**) running. The exo-tendon firstly emulates the double-actuation of hamstrings to store part of the kinetic energy during leg swing while providing hip with extension torque. As the force arm lever changes with hip and knee joint angles, the exo-tendon provides hip and knee joint with hip extension and knee extension torques during energy releasing phase. In this phase, the exo-tendon releases the stored energy to assist co-contraction of gluteus maximus and rectus femoris, and thus realize the phased modulation of hip–knee biomechanical energy.

As shown in Figure 2, inspired by the energy transfer function and double actuation of bi-articular muscles, we propose a hip–knee unpowered exoskeleton that exploits the bi-articular exo-tendon to modulate hip and knee joint powers in different gait phases. During the swing phase, the biarticular exo-tendon is stretched with knee extension and stores energy while passively providing extension torque for the hip joint. During the energy storage phase of the exoskeleton, the biarticular exo-tendon firstly imitates and enhances the double actuation of hamstrings, providing hip and knee joints with extension and flexion torque, respectively. Along with knee extension, the lever arm of the exo-tendon changes, and the assistive torque on knee joint changes from flexion torque to extension torque. In the following stance phase, the exo-tendon releases the stored energy and provides the hip and knee joints with extension torque. At this phase, the exo-tendon mimics and enhances the co-contraction of the gluteus maximus and rectus femoris. During the whole assistive process, the exoskeleton performs the combined functions of biarticular and mono-articular muscle groups in different gait phases, providing an external energy loop for the human body and efficiently realizing the energy transfer between multiple joints and gait phases.

### 2.3. Design of Hip–Knee Unpowered Exoskeleton

**Figure 3:**
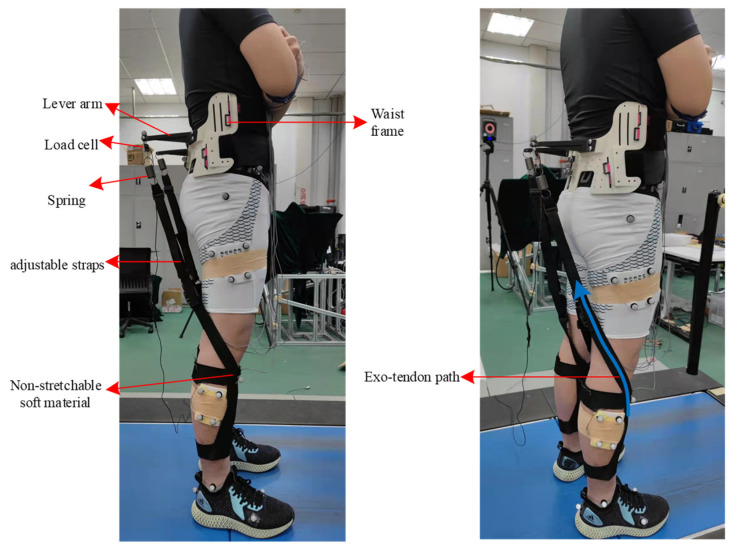
The design of multiarticular unpowered exoskeleton. The waist frame and shank frame were not connected with rigid links and joints to avoid misalignment between human joints and exoskeleton joints.

**Figure 4:**
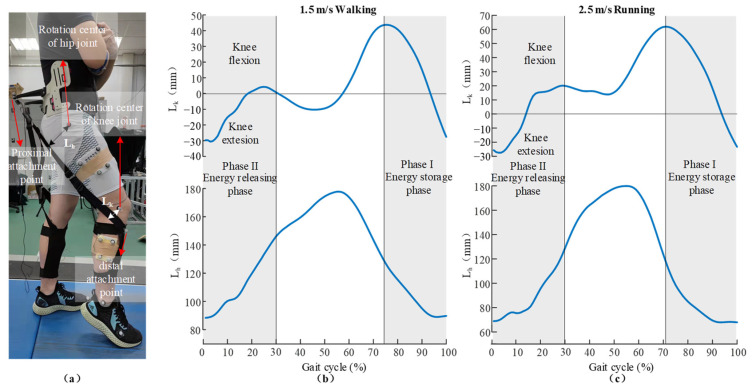
(**a**) The attachment points and real lever arms for hip and knee joints. (**b**,**c**) The length changes in hip lever arm (L_h_) and knee lever arm (L_k_) in gait cycles.

To realize the phased energy modulation in walking and running mentioned above, we designed the hip–knee unpowered exoskeleton (Figure 3). The exoskeleton consists of a waist frame, waist belts, lever arms, springs, adjustable straps and shank frames. The proximal attachment point of the spring is at the end of the lever arm. As the exoskeleton is designed to perform the combined functions of multiple muscle groups at different gait phases, the distal attachment of exo-tendon is designed to fix on the shank frame at the front of the lower leg. This design exploits the musculoskeletal structure of the human lower limbs, with the combined motion of the hip and knee joint to change the assistive torque on the knee joint. The specific process is shown in Figure 2, and the real lengths of the lever arms for the hip and knee joints are shown in Figure 4. During the swing phase, when the leg swings forward and the knee joint extends from the maximum flexion posture, the spring is stretched and provides the knee joint with flexion torque. Along with the knee extension in the late swing phase, the strap moves to the front of the rotation center of the knee joint, making the assistive torque change from flexion torque to extension torque on the knee joint. In the following stance phase, the spring releases the stored energy of the previous step with the hip joint extended and provides an extension assistive torque for the hip and knee joints. This design method, which utilizes the musculoskeletal structure and hip–knee angle changes to change the assistive torque of the exoskeleton to the knee joint, simplifies the exoskeleton mechanism and minimizes the mass of the exoskeleton, thus reducing the metabolic penalty of the exoskeleton at the distal end of the limb.

Modified orthotics methods and 3D printing methods were used to fabricate the waist part of the proposed exoskeleton for the best fit for the irregular surface of the human waist. The width of the left and right waist braces could be adjusted according to an individual’s body size. The waist frame was designed to attach to the human body with high stiffness points, which was reported in [8]. The waist frame was fixed to the waist with upper and lower waist straps, which prevented the waist frame from turning down during the assistive period. The shank brace was made of non-stretchable soft material to reduce the distal mass. The shank braces were fixed on the human lower leg by upper and lower straps, which exploited the irregular surfaces of the plantar flexors to prevent up and down displacement. As the waist and leg frames were segmented and not connected by rigid links, the freedoms of the hip and knee joints were not restricted by the exoskeleton. The adjustable straps, which are usually used on a backpack belt, were used to adjust the initial length of the exo-tendon. The length of the lever arm was 0.3 m. The spring stiffness of 3 kN/m was selected in the experiment based on the previous study of an exoskeleton for hip extension assistance [15]. The detailed exoskeleton mass distribution is presented in Table 1.

### 2.4. Experimental Protocol

Eight healthy male adults (175.4 ± 3.9 cm, 74.0 ± 8.9 kg, 25.4 ± 3.2 years) were recruited to participate in the experiment. The sample size was not predetermined by the statistical methods, and it was determined according to standard practice for walking and running research. The study was performed before the approval of the Chinese ethics committee of registering Clinical Trials, and all participants signed written informed consent.

The experimental protocol involved two main sessions: a walking and running habituation session (session I) and a walking and running testing session (session II). Session I and session II were performed on separate days to avoid muscle fatigue. In the walking and running habituation session, participants habituated themselves to the assistance of the proposed exoskeleton. The participants walked and ran with the exoskeleton at the speed of 1.5 m/s and 2.5 m/s, respectively, on the treadmill. Participants performed walking and running habituation trials twice, and each trial lasted 10 min. Participants could rest according to their needs between trials.

In session II, basic metabolic rate data were obtained in the standing-still trial. Then, the participants performed warm-up and testing trials. The warm-up and testing trials involved four conditions: walking with the exoskeleton (EXO_W), walking with no exoskeleton (NE_W), running with the exoskeleton (EXO_R) and running with no exoskeleton (NE_R). The walking and running trials were at the speed of 1.5 m/s and 2.5 m/s, respectively. Each warm-up trial lasted 2 min. After all warm-up trials were completed, the participants rested for 5 min and began the testing trials. In the testing experiments, each walking and running condition lasted 6 min and 5 min, respectively, which was consistent with the previous exoskeleton testing experiments. The participants were allowed to rest for 5 min between trials. The order of the four testing conditions was randomized. The metabolic rate, EMG, kinematic and kinetic data were measured. The experimental data of the last 2 min were analyzed.

### 2.5. Data Collection and Analysis

The indirect calorimetry system (Oxycon Mobile, CareFusion) was used to measure human oxygen consumption and carbon dioxide production, which are used to calculate metabolic rates through the Brockway formulation [40]. A reflective marker motion capture system (Vicon, Oxford Metrics;100 Hz) was used to record human lower-limb motion during walking and running. The EMG signals of the major flexors and extensors of the lower limb joints were measured via an electromyography system (SX230, Biometrics, Newport, UK). A treadmill instrumented with load cells (AMTI, Watertown, MA, USA, 1000 Hz) was used to measure the ground reaction forces during walking and running. The spring force was measured by the load cell (Forsentek, Shenzhen, China, 1000 Hz).

Joint angles, total joint torque and total joint powers were obtained from lower-limb motion data and ground reaction force data through inverse kinematics and inverse dynamics (Visual 3D, C-motion). In the EXO_W and EXO_R conditions, human joint torques and joint powers were obtained by subtracting the exoskeleton torque/power on the joint from the total joint torque/power. The exoskeleton torques on the hip and knee joints were calculated by multiplying spring forces and the level arm of the hip and knee joints, respectively (L_h_ and L_k_). The exoskeleton powers on the hip and knee joints were calculated by multiplying exoskeleton torques and the angular velocities of the hip and knee joints. The net metabolic rate of locomotion was obtained by subtracting the metabolic rate of standing still from the metabolic rate of walking/running. The EMG signals were rectified and low-pass filtered (fourth-order Butterworth, cut-off frequency 6 Hz) in MATLAB (MATLAB 2021b (9.11.0), Mathworks).

The exoskeleton torques, EMG signals, joint angles, joint moments and joint powers of each testing condition were divided into gait cycles, and average curves were calculated for each condition and each person. The EMG signals of each participant were normalized by dividing the maximum value of walking/running with no exoskeleton (NE_W/NE_R) conditions. The average EMG curves were averaged across participants for each condition. The means and standard errors of net metabolic rate, average moment, average peak joint angles, average peak joint powers and average EMG were calculated across participants. The means and standard errors of average EMG were calculated across participants over the whole gait cycle. A two-sided paired *t*-test was used to compare the experimental data of the EXO_W/EXO_R condition to the NE_W/NE_R condition, determining whether the assistance has a significant effect on metabolic rate, muscle activities, joint kinematics and joint kinetics.

## 3. Experimental Results

### 3.1. Metabolic Rate of Walking and Running

**Figure 5:**
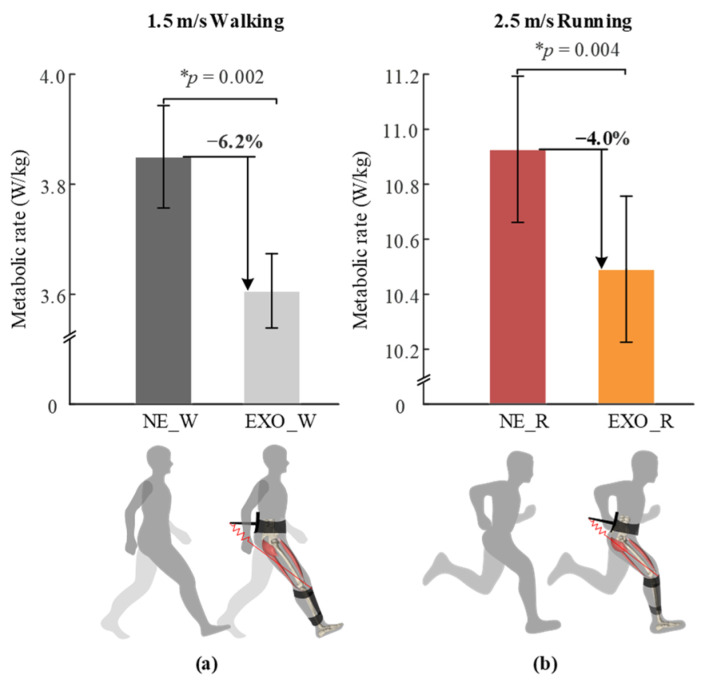
The average net metabolic rate in (**a**) walking and (**b**) running conditions. NE_W, EXO_W, NE_R and EXO_R represent walking with no exoskeleton, walking with exoskeleton, running with no exoskeleton and running with exoskeleton conditions, respectively. The asterisk (*) indicates the statistical difference between the two tested conditions using two-sided paired *t*-test.

As shown in Figure 5, the average net metabolic rates in the EXO_W, NE_W, EXO_R and NE_R conditions were 3.61 ± 0.07 W/kg, 3.85 ± 0.09 W/kg, 10.49 ± 0.27 W/kg and 10.93 ± 0.27 W/kg (mean ± s.e.m). The metabolic rates of walking and running were reduced by 6.2 ± 1.2% (mean ± s.e.m, *p* = 0.002, two-sided paired *t*-test) and 4.0 ± 1.0% (*p* = 0.004), respectively, compared to those of walking/running without the exoskeleton.

### 3.2. Muscle Activity

**Figure 6:**
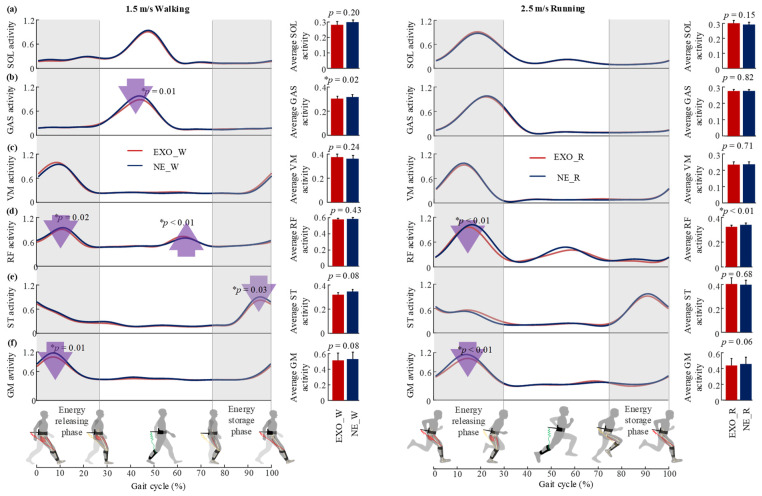
Changes in muscle activities in the tested walking and running conditions. (**a**–**f**) The activities of soleus (mono-articular muscle and ankle plantarflexor), gastrocnemius (GAS, biarticular muscle, ankle plantarflexor and knee flexor), vastus medialis (VM, major knee extensor), rectus femoris (RF, major hip flexor and knee extensor), semitendinosus (ST, major hip extensor and knee flexor) and gluteus maximus (GM, major hip extensor). The bar graphs represent the average muscle activities over the whole gait cycle in each experimental condition. The asterisk (*) indicates the statistical difference in peak muscle activities and average muscle activities between the tested conditions during walking and running using two-sided paired *t*-test.

As shown in Figure 6, the average muscle activities of target semitendinosus (ST) and gluteus maximus (GM) showed a decreasing trend (two-sided paired *t*-test, *p* = 0.08, *p* = 0.08) but not a significant reduction during the whole gait cycle compared to that in the NE_W condition. Specifically, the peak muscle activity of semitendinosus (ST) showed a significant reduction during the energy storage phase (paired *t*-test, *p* = 0.03), and the peak muscle activities of GM and rectus femoris (RF) decreased in the energy releasing phase (paired *t*-test, *p* = 0.01, *p* = 0.02), which may be the main reason for the decrease in the metabolic rate. Although the rectus femoris muscle peak EMG decreased significantly during the exoskeleton energy release phase, the average muscle activity of RF showed no significant change (*p* = 0.43) compared to NE_W, possibly due to the increase during the late stance phase (*p* < 0.01). Although the exoskeleton mainly assisted hip and knee movement, the peak and average muscle activity of the gastrocnemius muscle (biarticular muscle, main ankle plantar flexor and knee flexor) were significantly decreased (*p* = 0.01, *p* = 0.02), which was also the main factor for the reduction in the metabolic rate.

As shown in Figure 6, the average muscle activity of the target rectus femoris (RF) decreased with the assistance of the exoskeleton compared to that in the NE_R condition (two-sided paired *t*-test, *p* < 0.01). Specifically, the peak muscle activity of the RF was reduced during the energy releasing phase (*p* < 0.01). Although the average muscle activities of the vastus medialis (VM) and gluteus maximus (GM) did not show significant changes (*p* = 0.71, *p* = 0.06), the peak muscle activity of the GM was reduced during the energy releasing phase (*p* = 0.03, *p* < 0.01). The muscle activities of the gastrocnemius did not show a decrease, which was different from that of the walking condition.

### 3.3. Kinetics and Kinematics

**Figure 7:**
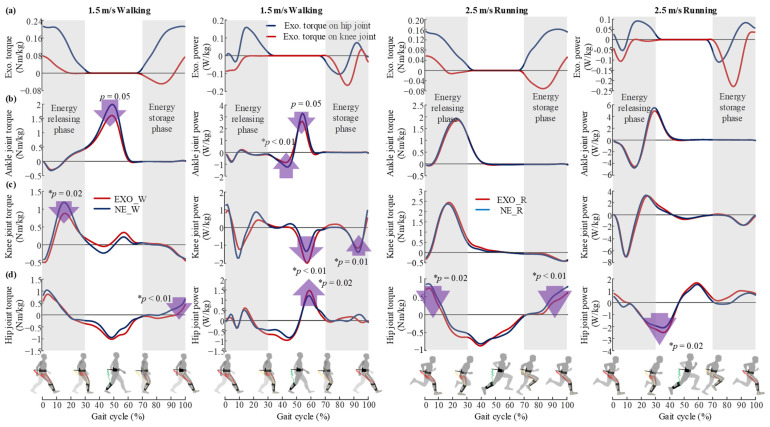
Exoskeleton mechanics and biological joint kinetics. (**a**) Exoskeleton torque and power on hip and knee joints in walking and running conditions. (**b**–**d**) Biological joint torque and power in walking and running conditions. The *p* values were the statistical comparison result of peak torque/power between NE (walking/running without exoskeleton) and EXO (walking/running with exoskeleton) conditions. The asterisk (*) indicates the statistical difference in peak joint torque and peak joint power between the tested conditions during walking and running using two-sided paired *t*-test.

As shown in Figure 7a, the peak exoskeleton torques on the hip and knee joints were 0.220 ± 0.031 Nm/kg and 0.077 ± 0.011 Nm/kg during walking. The peak exoskeleton torques on the hip and knee joints were 0.166 ± 0.031 Nm/kg and 0.057 ± 0.012 Nm/kg during running. During walking, the peak hip joint torque and knee joint torque decreased in the energy storage phase and energy releasing phase, respectively (*p* < 0.01, *p* = 0.02), which showed the same trend with the reduction in semitendinosus and rectus femoris activities. Although the exoskeleton did not provide direct assistance to the ankle joint, the biological ankle joint torque and power decreased during the push-off, which was the same trend as gastrocnemius activity. Unexpectedly, the peak positive power of the hip joint and the peak negative power of the knee joint increased (*p* = 0.02, *p* <0.01) during push-off, which were important factors affecting the assistant effect of the exoskeleton. The major changes in lower-limb biomechanics during running were concentrated in the hip joint torque and power. The peak hip joint torque decreased during both the energy storage phase and energy releasing phase (*p* < 0.01, *p* = 0.02). However, the peak negative power of the hip joint increased compared to that during running without the exoskeleton (*p* = 0.02).

**Figure 8:**
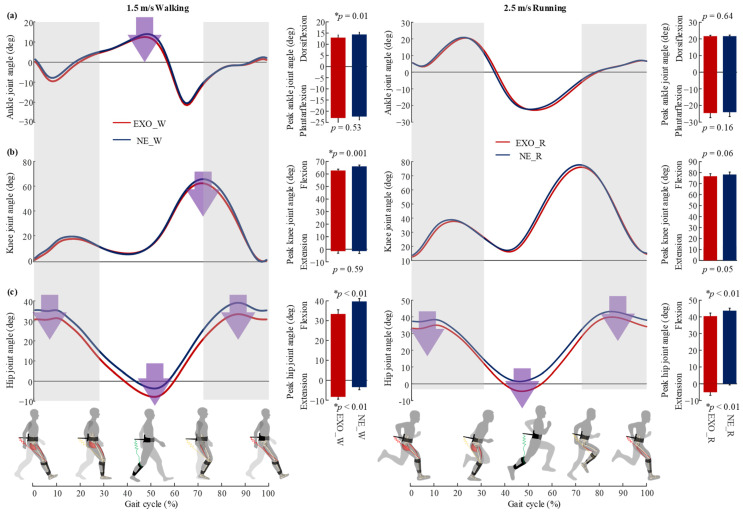
Changes in lower-limb kinematics in walking and running conditions. (**a**–**c**) The average curves of ankle, knee and hip joint angles across participants over the gait cycle. The bar graphs are the peak flexion and extension angles. The asterisk (*) indicates the statistical difference of peak joint angles between the NE and EXO during walking and running using two-sided paired *t*-test.

During walking (Figure 8), the peak joint angles showed significant changes with the exoskeleton assistance during walking except for the peak ankle plantarflexion angle (two-sided paired *t*-test, *p* = 0.53) and peak knee extension angle (*p* = 0.59). During running, the major changes were in the peak hip joint angles. The peak hip flexion angle decreased during the assistive interval (*p* < 0.01), and the peak hip extension angle increased (*p* < 0.01). The peak angles of ankle and knee joints showed no significant changes.

## 4. Discussion

This study aimed to demonstrate that it is possible to reduce the metabolic rate during both walking and running by modulating multiarticular power with an unpowered exoskeleton. Unlike the previous studies on autonomous hip exosuits [6] and unpowered hip exoskeletons [20], which provided assistance for single joints, the proposed hip–knee unpowered exoskeleton was designed to assist different muscle groups in different gait phases during both walking and running. The biarticular exo-tendons firstly assist the hamstrings to recycle part of the kinetic energy of the leg swing while passively providing the hip joint with extension torque. Then, the biarticular exo-tendons release the stored energy to assist both hip extension and knee extension in accordance with the co-contraction of the gluteus maximus and rectus femoris. Through the phased modulation of multiarticular power by the proposed exoskeleton, the metabolic rates of walking and running were reduced by 6.2 ± 1.2% and 4.0 ± 1.0%, which demonstrated the effectiveness of the proposed assistive method.

The most likely reason for the metabolic reduction during both walking and running might be the fact that the target muscle activities and biological joint moment were reduced with the assistance of the proposed exoskeleton (Figure 6 and Figure 7). During walking, the muscle activities of the semitendinosus, gluteus maximus and rectus femoris were reduced during energy storage phase and energy releasing phase, respectively (Figure 6), as were the peak hip joint moment and knee joint moment (Figure 7). These results indicate that the exo-tendon may partly replace the target muscles to perform mechanical work on joints, and thus reduce the muscle recruitment and biological joint moment. Another reason for the decrease in the walking metabolic rate was the significant decrease in gastrocnemius muscle activity and peak ankle moment and power during push-off (Figure 7). This was possibly due to the fact that the exo-tendon releases the stored energy to assist hip extension during the double stance phase. During the double stance phase, both ankle push-off actuated by the gastrocnemius and hip extension actuated by hip extensors together propel the human body to move forward [22,41]. As the stored energy of the exo-tendon is released to assist hip extensors, the trade-off between ankle push-off and hip extension may be changed with less effort needed by the gastrocnemius [42]. Unexpectedly, the assistance of the exoskeleton also caused an increase in the muscle activity of the rectus femoris and hip joint power, which was a negative effect. This was possibly due to the fact that the exo-tendon stores part of the kinetic energy of the leg swing, which is partly provided by the hip flexors [43]. During running, the participants showed different responses to the assistance of the exoskeleton from that of walking. The reduction in the peak muscle activities of the rectus femoris and gluteus maximus and biological hip joint moment may be the main reason for the metabolic reduction during running. The muscle activities of the gastrocnemius, the peak biological knee joint moment/power and the peak biological ankle joint moment/power also did not show significant changes during push-off, which was also different from that of walking.

The separate design of the shank frame and waist frame and the lightweight exoskeleton structure are important factors affecting the assistance effect. The separate design of the exoskeleton avoided the resisting moment caused by misalignment between the exoskeleton joints and human joints, which was a key factor preventing previous rigid exoskeletons from reducing the metabolic rate. We developed the shank frame using soft materials to reduce the exoskeleton mass distal to the human trunk and thus reduce the metabolic penalty, especially during running. The metabolic penalties of the total exoskeleton mass were only 2.48 W and 5.54 W during walking and running, respectively (metabolic penalty coefficients × exoskeleton mass, summarized in supplementary of [6]).

Compared with previous multiarticular unpowered exoskeletons [14,15,29], we found a new way to modulate multiarticular power and thus reduce the metabolic rate of both walking and running. Previous multi-articular unpowered exoskeletons mainly had designs inspired by the musculoskeletal structure of a certain biarticular muscle group, such as the gastrocnemius [29], rectus femoris or hamstrings [14]. Although it has been proved that the metabolic rate during walking can be reduced through the assistance of energy transfer between joints and gait phases, it has not been proved that these methods can be applied to running. In this paper, we expanded the assistive method. The proposed exoskeleton assists different biarticular muscle groups to realize efficient energy recycling between gait phases and energy transfer between joints. The experimental results during running also support the hypothesis that recycling part of the kinetic energy during the swing phase to assist other muscle groups is an effective way to reduce the metabolic rate of running [44].

Although the experimental results demonstrated that the metabolic rate of both walking and running can be reduced with the assistance of the proposed exoskeleton, we acknowledge that there are still several limitations in this study. First, the proposed exoskeleton provided assistance for multiple joints; however, the metabolic reduction was less than that in previous work on hip unpowered exoskeletons [16,20] and autonomous hip powered exosuits [6] which only provided assistance to the hip joint. The most likely reason for this is that we did not optimize exoskeleton assistive parameters, such as spring stiffness and the ratio of exoskeleton torque on hip and knee joints. Future work may include parameter sweeping experiments to optimize spring stiffness and the lever arm ratio of the hip and knee joints to determine whether the metabolic rate can be further reduced. We will also test other spatial–temporal parameters, such as fluctuations in center of mass (COM) and stride length, to determine how assistance affects human natural gait.

## 5. Conclusions

In this paper, we proposed an unpowered exoskeleton to modulate multiarticular energy during both walking and running, based on the study of the double-actuation of hamstrings and the co-contraction of mono-articular (gluteus maximus) and biarticular (rectus femoris) muscles. The exo-tendon firstly assists hamstrings in recycling part of the kinetic energy of the leg swing while simultaneously providing knee flexion and hip extension. The assistance torque for the knee is changed from knee flexion torque to knee extension torque with changes in the hip and knee angles. Then, the exo-tendon releases the stored energy to assist both hip extension and knee extension during the stance phase. The metabolic rate can be reduced by 6.2% and 4.0% during walking and running, respectively, which demonstrates the effectiveness of exoskeleton assistance. The design method may inspire the design of future multiarticular exoskeletons for assistance in multiple gaits. The experimental results of muscle activities and kinetics also provide an insight into different human responses to multiarticular assistance between walking and running.

## Figures and Tables

**Table 1:** The mass distribution of unpowered hip–knee exoskeleton.

Segment	Mass (g)
Wasit frame (including lever arm)	340
Springs (×2)	110
Shank frame (×2)	100
Total mass (biarticular sum)	550

## Data Availability

The data that support the findings of this research are available from the corresponding author, Wenbin Chen, upon reasonable request.
